# Angiotensin II-receptor blockers in bicuspid aortic valve patients with aortic dilation: Groping in the dark

**DOI:** 10.1016/j.ijcchd.2022.100412

**Published:** 2022-07-19

**Authors:** F. Meccanici, J.W. Roos-Hesselink

**Affiliations:** Department of Cardiology, Erasmus University Medical Centre, Rotterdam, the Netherlands

Aortic aneurysms represent a silent yet dangerous disease. Progressive dilation of the aorta confers a risk of rupture or dissection, which has an estimated incidence of 4.6–6.0 per 100.000 annually in the general population [1,2]. In case of an aortic dissection or rupture, it is often too late and mortality is high, estimated at 21–24% in type A aortic dissections, if patients even reach the hospital [3,4]. In specific patient groups aortic dilation is common even at a young age, such as patients with connective tissue disease. Bicuspid aortic valve (BAV) is a common congenital heart defect with a prevalence of 0.5–0.8 per 1000 live births [5,6] and these patients often suffer from aortopathy. A recent population-based study showed that aortic dilation was present in 32% of the newborns with BAV [6]. During their lifetime the risk of aortic dissection in patients with BAV is around 3.1 per 10.000 patient-years and 8 times higher than in the general population [7]. In order to prevent aortic dissection or rupture, preventive aortic surgery can be performed. However, this is a major intervention with associated complications and the optimal timing of the intervention is debatable. Therefore other less invasive treatment methods to decelerate aortic growth, such as medical therapy, are of great importance.

In clinical practice, beta blockers (BBs) are recommended in syndromic aortic disease as therapy to slow down aortic dilation and reduce wall shear stress, although the effects have not been confirmed in clinical trials [8]. Both the transforming-growth factor-β (TGF-β) pathway and the renin-angiotensin system might play an important role in aneurysm formation [9]. Therefore, TGF-β neutralizing antibodies or angiotensin receptor blockers (ARBs), which also have TGF-β blocking capabilities, are considered potential therapeutic compounds. In patients with Marfan syndrome (MFS), known for its aggressive aortopathy, a meta-analysis of clinical trials showed that ARBs might be associated with slower progression of aortic dilation, although the evidence is not overwhelming [10]. In this light, BBs or ARBs might also decline progression of aortic dilation in young patients with BAV. However, the evidence for effective treatment is scarce. Two small retrospective cohort studies have provided some evidence in pediatric BAV patients: Warren et al. (n = 88) found an association of BBs with slower aortic growth [11], whereas Flyer et al. (n = 45) found a reduction in aortic growth for losartan (ARB) and atenolol (BB) [12].

In this journal Mariucci et al. describe the results of a retrospective cohort study on 191 pediatric patients with normally functioning BAV, examining the aortic diameters serially from 2005 to 2021. The authors have described two patient groups: 1) patients with normally functioning BAV not receiving medical therapy (n = 175) and 2) patients with normally functioning BAV with progressive aortic dilation, in which medical therapy was initiated (n = 30). During a mean follow-up of 3.7 ± 2.7 years the proportion of aortic dilation remained stable in the first group (46.3% versus 52.6% p = 0.280). In 92/175 patients new onset or progressive aortic root dilation was observed, which was associated with BAV with a raphe (p = 0.037) and mild aortic regurgitation (p < 0.001). In the second group of BAV patients receiving medical therapy (n = 30), losartan was the most frequently described drug (n = 28), followed by atenolol (n = 1) and enalapril (n = 1). The authors concluded that after a mean follow-up of 3.3 ± 2.3 years, aortic diameters did not change significantly. In 14/30 patients, the aortic diameters before and after treatment were evaluated, and no significant difference was observed in aortic z-scores.

Although the authors have collected valuable data on an understudied subject, some comments can be made on this study and its interpretation. In the statistical analysis, the authors have had some missed opportunities to study this relevant data. Despite the fact that they have collected data on the aorta serially, they have dichotomized aortic growth and used only the baseline and follow-up measurements. These measurements would rather be analyzed with linear mixed models, taking into account the repeated measurements, just like the analysis by Flyer and colleagues [12]. It might be questioned whether the same results would be obtained by using these models. Additionally, the mean aortic z-scores were compared between groups of patients, rather than a matched comparison of baseline with follow-up in each patient. The latter would have been preferred as the follow-up diameters were likely correlated with the baseline diameter within each patient.

In this study the indication for prescribing medication was not completely clear. A selection bias was introduced by the prescription of medication to the patients with progressive aortic root dilation, on the other hand it reflects a true presentation of clinical practice. Also, unfortunately only on 47% of the patients the aortic diameters before initiation of the medication was known. Therefore the conclusion of the authors that the use of medical therapy did not affect the rate of aortic dilatation in patients with progressive aortic dilatation, is perhaps too strong. A randomized clinical trial setting would be more optimal to study the causal effect of ARBs with limited selection bias.

As the authors have reflected on in their discussion, their study population differs significantly from the other studies [11,12] reporting on this subject. In these studies only patients with associated complex congenital heart disease were excluded. By excluding patients with aortic valve dysfunction in the study by Mariucci et al., a more selected study population is chosen. However, the authors might have missed many patients with aortic dilation, as aortic valve disease is associated with aortic dilation in patients with BAV [7]. It could be possible that the effect of medication on aortic dilation is greater in the patients with a more severe form of BAV disease, as protective effects in the other studies were suggested [11,12]. On the other hand, one might argue the safety of prescribing these agents to patients with severe aortic stenosis, as the afterload will be lowered.

Moreover, blood pressure lowering is considered one of the mechanisms of ARBs in declining aortic growth. It might have been interesting to study the effect of ARBs on blood pressure and heart rate of these patients, to gain more insight on the working mechanism. Unfortunately, blood pressure nor heart rate was collected in this study, or not reported.

Furthermore, some questions remain on the choice of medication. Nearly all patients (93%) received losartan. As the effects of ARBs and BBs might differ, these would rather be described separately. Arguably, the dosage of ARBs in this study was not sufficient to observe a treatment effect. The mean dosage of ARB used in this study was 0.5 ± 0.2 mg/kg/day, compared to a median of 0.95 (0.64–1.45) in Flyer et al. [12]. It has been proposed that administration of ARBs in the range to lower blood pressure is not satisfactory in normalizing TGF-β overexpression effectively [9].

Interestingly, gender differences exist in the pharmacokinetics and pharmacodynamics of cardiovascular medication [13]. In the future, it might be of interest to study male-female differences in the dosage and effectiveness of ARBs and BBs concerning aortic dilation in BAV.

Accordingly it seems that the topic of prescribing medication in pediatric BAV patients remains unresolved and in need of further study. The authors pose clinical trials as the best option for further research, however in real life this might be difficult to organise. In the clinical trial register of the National Library of Medicine two studies can be found on the subject of medication to prevent progression of aortic dilation in bicuspid aortic valve aortopathy, and both studies have been terminated early due to poor patient recruitment. As aortic growth is slow and the incidence of aortic events low, a very long follow-up would be needed with many patients to study the effect of an intervention. Possibly, a large prospective international registry with measurements before and after treatment initiation, might be a more pragmatic study design.

More importantly, it can be questioned whether prescription of medication in pediatric BAV patients is desirable. These young patients are already hampered in daily activities such as sports and have the burden of regular doctor visits. Life-style interventions such as lowering salt intake, physical activity or caloric restriction, might be more valuable in preventing aortic dilation and improving their overall cardiovascular health, including their aorta. In MFS mouse models mild to moderate dynamic exercise has shown to reduce aortic growth compared to sedentary MFS mice [14]. Additionally, diet seems to play an important role: a recent study on older adults with obesity showed that caloric restriction in addition to exercise expedited greater improvements in proximal aortic stiffness compared to exercise alone [15].

For now, the evidence to prescribe medication in pediatric patients with bicuspid aortic valves and aorta dilatation is still not convincing, although the study of Mariucci et al. has provided valuable insights. Research on medical treatment for aortic dilation, but also on possible other interventions for instance on life style remain of great importance, as aorta pathology poses a cardiovascular burden already at young age in patients with bicuspid aortic valves.

## Declaration of competing interest

The authors declare that they have no known competing financial interests or personal relationships that could have appeared to influence the work reported in this paper.
